# Molecular Identification of Selected Cervid Helminths in Supplementarily Fed European Bison Population

**DOI:** 10.1155/2024/2600633

**Published:** 2024-11-09

**Authors:** Magdalena Świsłocka-Cutter, Rafał Kowalczyk, Anetta Borkowska, Tomasz Kamiński, Marta Kołodziej-Sobocińska

**Affiliations:** ^1^Department of Zoology and Genetics, Faculty of Biology, University of Białystok, Ciołkowskiego 1J 15-245, Białystok, Poland; ^2^Mammal Research Institute, Polish Academy of Sciences, Stoczek 1, 17-230, Białowieża, Poland

**Keywords:** cervids, endangered species, endoparasite richness, helminths, molecular markers, winter supplementary feeding

## Abstract

**Background:** Wild animals often suffer from infections with multiple species of parasites simultaneously. The exchange of parasites between different host species is common in nature and often involves intermediate hosts or sharing space such as pastures or watering holes. Supplementary feeding, leading to large aggregations of individuals, can have several adverse effects on wild ungulate populations, despite being a widespread management practice. One such effect is an increased risk of parasitic infections, particularly in social animals. We quantified the prevalence of selected helminths typically found in cervids, in samples of the European bison faeces, using molecular methods, and compared endoparasite species richness between supplementarily fed and nonfed European bison herds in the Białowieża Primeval Forest, NE Poland.

**Methods:** Using the diagnostic PCR method, we analysed the faecal samples for molecular markers of nine parasite species which are typically found in cervids: moose, red deer, and roe deer.

**Results:** All analysed samples tested positive for at least one parasite species, and the average number of parasite species per sample was 3.2. The most prevalent parasites were gastrointestinal nematodes: *Ostertagia leptospicularis* and *Ostertagia antipini*, found in 89.2% and 50.6% of the European bison faecal samples, respectively. We found significant differences in the prevalence of four parasite species between supplementarily fed and nonfed European bison herds. Co-occurrence analysis showed that most of the associations between parasite species pairs were random.

**Conclusion:** Management practices, such as supplementary feeding, can influence the spread of parasite infections in social mammals. This study also promotes the application of molecular methods for noninvasive parasitological monitoring of wildlife populations of endangered ruminant species sharing resources with other ungulates.

## 1. Introduction

Parasites can influence the dynamics of wild ungulate populations through their negative impacts on the body condition and fecundity of hosts [1, 2]. They are often a threat to endangered species, whose health is a key focus of conservation programs [3, 4]. High population densities, human-driven changes to natural habitats, and the spread of invasive species may aid the transmission of parasites between individuals in the wild [5]. Among the factors that contribute to the increase in population density of protected species are management practices such as supplementary feeding. Despite the positive impact on the condition of animals, it can also have some negative effects on the population, mainly related to excessive concentration of animals, which leads to disease transmission [6].

One such animal is the European bison (*Bison bonasus* L., 1758), which is the largest terrestrial mammal in Europe. It was extirpated in the wild at the beginning of the 20th century and later restored from captive survivors [7]. The species has recently been assessed by the IUCN Red List of Threatened Species as near threatened [8]. Now, there are over 8200 free-living European bison worldwide, of which nearly 2400 individuals are in Poland. The European bison population in the Polish part of the Białowieża Primeval Forest (BPF) was restored in the wild and is the largest one; it currently consists of 829 individuals [9]. The population is widespread in the forest and surrounding mosaic areas covering over 800 km^2^ [10]. During winter (December–March), the European bison are supplementarily fed in several feeding stations to provide the herds with forage when food is scarce in forest habitats and to mitigate their migration out of BPF. Another goal of this practice is to reduce potential damage to crops [11]. However, it leads to large aggregations of the European bison in fixed locations and increased contact rates between individuals, which enables interindividual transmission of parasites such as the blood-sucking nematode *Ashworthius sidemi* [6, 12] and coccidia [13].

Multiple infections of a host with different parasite species are frequent in the wild, and the exchange of parasites between different host species is also common in nature [14]. A good example is the case of white-tailed deer (*Odocoileus virginianus*) and moose (*Alces alces*) in North America. White-tailed deer are the definitive host for meningeal worm (*Parelaphostrongylus tenuis*), which causes neurologic disease and increased mortality in moose. The moose population has decreased in the areas with high deer density and the highest concentration of parasite larvae in deer faeces [15]. For many parasites, transmission depends on host population density but most often requires sharing pastures, waterholes, or intermediate hosts [15, 16].

Three native species of cervids are found in BPF; red deer (*Cervus elaphus*) are the most numerous, followed by roe deer (*Capreolus capreolus*) and moose (*Alces alces*) [17]. They share the same habitats and resources with the European bison; thus, the risk of host crossinfection exists and poses a major problem for the conservation of this threatened species.

The European bison populations have been closely monitored in several parasitological studies using both coprological methods and postmortem examinations. A total of 88 parasite species were described, including the most prevalent nematodes and species of protozoa, followed by mites, trematodes, cestodes, and insects [18, 19]. The most numerous group of parasites are helminths (51 species) with 17 parasite species specific to Cervidae: one fluke and 16 species of nematodes. Most of them were transferred to bison from deer. The occurrence of nematode *Spiculopteragia asymmetrica* was recorded in bison for the first time in the 1990s, and blood-sucking abomasal nematode *A. sidemi* (first found in 2000) can be correlated with the increase in the number of red and roe deer in BPF and contact of the restituted population with deer. *Parafasciolopsis fasciolaemorpha*, *Nematodirella alcidis*, and *Trichostrongylus capricola* were observed as new bison parasites after the restoration of the moose population in north-eastern Poland. The changes were observed not only in species richness but also in the prevalence and intensity of infection, for example, *Ostertagia* sp. [19, 20].

Noninvasive endoparasite monitoring in the European bison populations in Poland, including BPF, has been achieved in the past through coprological methods such as the identification of eggs, oocysts, larvae, or tissues of distinct endoparasite species excreted in faeces [13, 18, 21]. Due to the overlapping morphological characteristics in some of the development stages, traditional macroscopic methods may be error-prone and not very sensitive as they generally determine identity to the genus level, for example, *Ostertagia* sp. or *Haemonchus* sp. [18, 22]. Species-level identification of endoparasites using conventional methods typically requires adult specimens. Collection of parasite specimens in postmortem examinations of wild mammals is limited by the availability of carcasses, especially in the case of fully protected and endangered species. The bison population in BPF has been regulated since the 1970s through culling or translocation [23]; however, only 41 postmortems, which included parasitological examination, have been performed on wild-born and free-living individuals between 2002 and 2016 [4]. The molecular identification of endoparasites offers a good alternative to traditional parasitological approaches. Molecular methods are more sensitive, are less subjective, and show better taxonomic resolution than egg and larva counts, which makes them ideal for assessing parasite species richness and noninvasive monitoring of endoparasite fauna in a natural host population [24, 25].

The objectives of this study were to (1) perform a methodology for noninvasive molecular monitoring of the helminths in the European bison, (2) quantify the prevalence of selected helminths typically found in cervids in the noninvasively collected faecal samples of the European bison using molecular methods of parasite species identification, (3) compare endoparasite species richness between supplementarily fed and nonfed European bison herds, and (4) examine parasite association between coinfecting parasite species in the European bison from the BPF.

## 2. Material and Methods

### 2.1. Ethics Statement

No live animals were used in our survey. Samples of the European bison's faeces were collected in the wild in the BPF, NE Poland, in accordance with the local regulations.

### 2.2. Study Area, Sample Collection, and Laboratory Analyses

**Figure 1:**
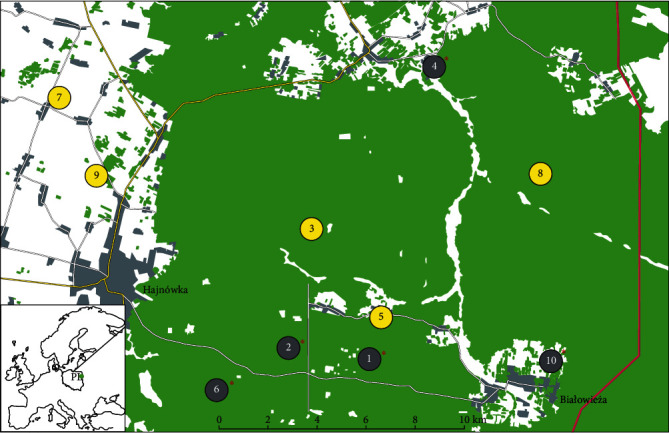
Locations where faecal samples of supplementarily fed (grey) and nonfed (yellow) European bison herds were collected in winter in the BPF, NE Poland. ⁣^∗^Locations of winter feeding stations with supplementary food.

The study was conducted in the BPF, NE Poland (52°29⁣′–52°37⁣′ N, 23°31⁣′–24°21⁣′ E; Figure 1). It is one of the best preserved European lowland forests recognised as a UNESCO World Heritage Site. The Polish part of BPF covers 635 km^2^ of mosaic habitats and tree stands (REF) [26, 27]. The European bison population in BPF has been extensively monitored and studied since the species' restoration and reintroduction into the wild in the 1950s [23]. In summer, European bison usually utilise forest habitats, while in winter, they aggregate in larger herds and migrate to supplementary feeding sites within the forest or to open habitats (meadows and farmland) surrounding the BPF [10]. Fresh European bison faecal samples were collected from 10 European bison herds around the feeding sites within the forest or in open habitats outside the forest between the 14th and 21st of February 2020 (Figure 1). The number of collected samples in analysed herds ranged from four samples (Herd No. 5) to 12 samples (Herd Nos. 4 and 9). The samples were divided into two groups: (1) collected from supplementarily fed herds (Herds 1, 2, 4, 6, and 10; *n* = 43 samples) around feeding sites and (2) collected from herds staying out of the feeding sites in winter (Herds 3, 5, and 7–9; *n* = 40 samples). This division was based on the results of previous studies on the relation between winter densities, herd size, and winter home ranges of European bison and the infection rate of blood-sucking nematode *A. sidemi*, where fed and nonfed herds differed in the parasite dynamics [6, 12, 27, 28].

Faecal samples were cooled and stored at −20°C, to preserve parasites until molecular identification could take place. DNA was extracted and purified using the DNeasy Blood and Tissue Kit (Qiagen, Hilden, Germany) from ~0.025 g of faecal samples, according to the manufacturer's protocol.

Initially, 16 randomly chosen DNA samples of *Bison bonasus* were screened for selected cervid parasite presence using 16 published or newly designed primer pairs (Table S1) for molecular identification of tapeworms: *Moniezia benedeni* (Cestoda, Anoplocephalidae); *Echinococcus granulosus*; and larval form of *Taenia hydatigena*, called *Cysticercus tenuicollis* (Taeniidae); flukes: *Paramphistomum cervi* (Trematoda, Paramphistomidae) and *P. fasciolaemorpha* (Trematoda, Fasciolidae); cerebrospinal nematodes: *Elaphostrongylus alces*, *Elaphostrongylus cervi*, and *Parelaphostrongylus tenuis* (Nematoda, Protostrongylidae), lungworms: *Dictyocaulus capreolus* and *Dictyocaulus cervi* (Dictyocaulidae); and gastrointestinal nematodes: *Ostertagia antipini* and *Ostertagia leptospicularis* (Trichostrongylidae) and two blood-sucking nematodes *A. sidemi* and *Haemonchus contortus* (Trichostrongylidae), as well as *Oesophagostomum venulosum* (Chabertiidae) and *Setaria tundra* (Setariidae). Each forward primer was end labelled with one of the following fluorescent dyes: VIC, FAM, NED, or PET (Life Technologies Inc.), and optimised for resolution on an ABI 3130 Genetic Analyzer. PCRs were conducted in three multiplex panels to avoid overlap between analysed parasite gene fragments labelled with the same dye (S1 Table). PCR reactions were performed with ~25 ng genomic DNA, 1.7-*μ*L Qiagen Multiplex PCR Master Mix (1x), 0.3-*μ*L mix of species-specific primers, and 1-*μ*L Qiagen RNase-free water in a 5-*μ*L reaction volume (Qiagen, Germany). The PCR amplification was performed in a Labcycler Gradient (SensoQuest, Goettingen, Germany) with the following profile: initial denaturation at 95°C for 15 min, followed by 38 cycles consisting of denaturation at 94°C for 30 s, annealing at 57°C for 90 s, extension at 72°C for 60 s, and final extension step at 60°C for 30 min.

Finally, in the absence of readings for seven species of endoparasites in 16 randomly chosen DNA samples, all analysed faecal samples were molecularly tested for nine species of parasites confirmed in two multiplex PCR panels: panel I: *P*. *fasciolaemorpha*, *P*. *cervi*, *O*. *antipini*, *O*. *venulosum*, and *H*. *contortus*, and panel II: *M*. *benedeni*, *T*. *hydatigena*, *A*. *sidemi*, and *O. leptospicularis*.

The products of amplification reactions obtained through molecular analyses were then separated by size by capillary electrophoresis (3130 Genetic Analyzer, Applied Biosystems) using the GeneScan 500 LIZ standard. Then, the electromorphs, read as coloured peaks (VIC, FAM, NED, or PET) obtained for the analysed DNA markers, were assigned to endoparasite species with GeneMapper 4.0 (Applied Biosystems). To ensure the reliability of the molecular analyses, we randomly repeated the amplifications of all selected nine endoparasite-specific DNA fragments for 10% of the samples and compared the results with those obtained from the first amplification.

### 2.3. Statistical Analyses

The proportion of infected samples, that is, the prevalence of the nine endoparasite species, was determined for all samples and each feeding group (supplementarily fed and nonfed herds). Ninety-five percent confidence intervals (95% CI) for prevalence based on the Wilson score interval were calculated using OpenEpi (http://www.openepi.com/Proportion/Proportion.htm). The chi-squared test in OpenEpi (http://openepi.com/TwobyTwo/TwobyTwo.htm) was performed to test the statistically significant differences in the prevalence of a particular parasite species between supplementarily fed and nonfed herds. The number of endoparasite species in each faecal sample (parasite species richness) was also calculated. The Mann–Whitney *U* test (TIBCO Software Inc. 2017) and chi-squared test for trend were conducted to compare parasite species richness between fed and nonfed herds (https://www.medcalc.org). *p* values smaller than 0.05 were considered significantly different. A probabilistic model of species co-occurrence [29], implemented in the “cooccur” package 1.3 version [30] of the statistical computing language R [31], was applied to detect pairs of parasite species that share hosts with different frequencies than could be expected, if their occurrence rates were independent of each other. Pairs of species with the expected co-occurrence lower than 1 were removed from the analysis. If a species pair is not classified as positive or negative, then it could be truly randomly distributed or unclassifiable due to low statistical power. Truly random associations are those that do not deviate from their expected co-occurrences by more than 0.1 × the total number of sites [29].

## 3. Results

**Figure 2:**
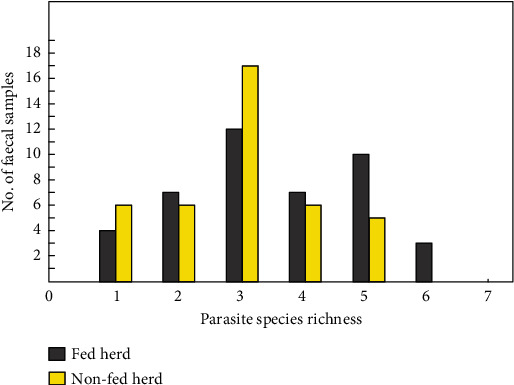
Parasite species richness in the European bison faecal samples obtained from winter supplementarily fed herds (grey) and nonfed herds (yellow) from the Białowieża Primeval Forest.

The molecular analyses showed that all of the European bison faecal samples (100%, CI: 95.58–100) were positive for at least one parasite species, with the maximum of six in a single sample. Most commonly, DNA of three endoparasite species was found: *O*. *leptospicularis*, *O*. *antipini*, and *A. sidemi* (34.9% of samples; Figure 2), and the presence of six different parasite species was detected in 3.6% of examined samples.

Samples collected from nonfed herds tested positive for up to five parasite species, and no differences both in parasite species richness (median = 3, range 1–6, *U* = 695, *p* >0.05) and in the proportion of samples infected by different numbers of parasite species (chi-squared test for trend, *χ*^2^ = 3.35, *p* > 0.05; Figure 2) were found in comparison to those from supplementarily fed herds. However, three parasite species were present significantly more often in fed than nonfed herds: *O. venulosum*, *P. fasciolaemorpha*, and *M. benedeni*, while *P. cervi* showed a higher prevalence in nonfed herds (Table 1).

**Figure 3:**
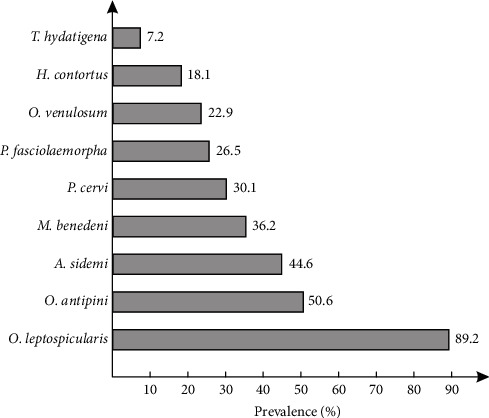
Prevalence of endoparasite species diagnosed using molecular methods from 83 faecal samples of the European bison collected from the Białowieża Primeval Forest.

The most prevalent parasite species were the gastrointestinal nematodes *O*. *leptospicularis* and *O. antipini* which were identified in 89.2% and 50.6% of tested samples, respectively (Figure 3).

The most frequent parasite, *O*. *leptospicularis*, was found with a prevalence of 85% in nonfed herds and 93% in fed herds (Table 1). This helminth was found in all studied European bison herds, with prevalence ranged from 66.7% (Herd No. 9) to 100% (Herd Nos. 6, 7, 8, and 10; Table S2). The least common parasite species was the tapeworm *T. hydatigena*, which was found in 5% of samples from nonfed herds and 9.3% of samples from fed herds (Table 1). The parasite was found in four European bison herds with the proportion of infected samples ranging from 8.3 (Herd No. 4) to 28.6 (Herd No. 7; Table S2).

The analysis of co-occurrence indicated that most of the classifiable endoparasite species pairs (76.2%) showed random associations. Only three positive and two negative associations between 36 parasite species pairs were found. Statistically significant positive associations were noted for *O*. *venulosum*–*T*. *hydatigena* (*p* = 0.0019), *O*. *venulosum*–*P*. *fasciolaemorpha* (*p* = 0.0048), and *H*. *contortus*–*P*. *cervi* (*p* = 0.0075). Parasite pairs *O. leptospicularis*–*P*. *cervi* (*p* = 0.0181) and *O*. *antipini*–*H*. *contortus* (*p* = 0.0424) were present in the same sample less frequently than expected.

## 4. Discussion

Multihost pathogens are a major wildlife conservation problem, and their distribution in wild animal populations is still not well recognised [32–35]. Almost half of the studied wild ungulates individuals (red deer, mouflon, Iberian ibex, and fallow deer) in southeastern Spain was infected with bronchopulmonary nematodes representing seven different species [36]. These lungworms have a direct impact on domestic and wild ruminants, negatively affecting their health and fitness. Our molecular survey allowed us to describe the prevalence of selected Cervidae helminths in the European bison population, where some herds are experiencing supplementary feeding practice. Noninvasive molecular monitoring of parasitofauna typically found in cervids was successfully tested in the European bison for nine species of endoparasites. It was the first such investigation in this species.

Although wild ungulate populations are intensively monitored across Europe, we know little about the influence of parasites on host population dynamics, and there is no systematic, long-term monitoring of parasite diversity and parasite loads. Such monitoring is in part hampered by the short duration of projects and the cost of assay methodologies that are characterised by high sensitivity and good taxonomic resolution [25]. Molecular methods for the detection of DNA of specific parasite species are necessary to obtain reliable and accurate results [4, 37–39] and are increasingly frequently used to monitor parasite biodiversity in wildlife populations [40]. Compared to traditional microscopy-based methods, they are more sensitive and provide better taxonomic resolution for the detected taxa [41], which is particularly important in research where analysed material consists of noninvasively collected material, for example, fresh faeces. One shortcoming of the molecular approach to parasitological research is that it is a purely qualitative method, not a quantitative one. This approach does not allow us to determine the intensity of infection, which along with factors such as the host's age or state of its immune system often has a much greater impact on the infected host than the richness of endoparasite species [42, 43]. However, molecular analyses of gastrointestinal nematode parasites in Norway moose populations allowed for a better estimation of the diversity and the range of species infecting a given individual compared to quantified parasitological techniques based on egg and larva counting [25]. Molecular methods fill the gaps in traditional morphological assays and, together with ecological data on a given population, allow us to accurately assess the condition of the studied populations.

Parasitic infections have been extensively investigated in the European bison population in Poland for decades, leading to the identification of a total of 88 parasite species [18, 19]. Almost all parasites affecting bison are polyxenic, have a large range of hosts, or are shared with other wild ungulates or cattle [23, 44]. The exchange of parasites between different host species is common in nature but most often requires sharing space such as pastures, waterholes, or intermediate hosts. This survey is the first attempt at complex molecular identification of selected endoparasite species in the biggest European bison population in Poland, inhabiting the BPF (NE Poland). Based on genetic analysis, the presence of nine endoparasite species was confirmed across the collected 83 samples of the European bison faeces, and these results are similar to the earlier studies, in which the same parasite species have been identified using morphological methods [18, 19].

Some of the European bison parasites detected in this study are typically found in Cervidae (for *O*. *antipini*, *O*. *leptospicularis*, *A*. *sidemi*, and *P*. *fasciolaemorpha*) but also in cattle (for *M*. *benedeni*, *P*. *cervi*, *O*. *venulosum*, and *H*. *contortus*) and Canidae (for *T*. *hydatigena*) [19]. The sharing of resources by Cervidae, cattle, and the European bison is undoubtedly a threat to the health of this endangered species. It is noted that the helminth and arthropod fauna of free-living European bison is richer than of that from captive populations, due to the acquisition of new species of parasites from Cervidae [45].

In the present study, the most prevalent endoparasite species found in the European bison that are typically present in other ungulates were gastrointestinal nematodes *O*. *leptospicularis* and *O*. *antipini*. They were detected with a very high prevalence, reaching 90% and 50%, respectively, which is an interesting result because these parasites were acquired by the European bison from Cervidae during the second half of the 20th century [46]. It is worth noting that these parasite species occur wherever moose is present and form the associated parasite–host system with this cervid [47], the population of which has been restored in eastern Poland in recent years [48]. A parasite that is typical for Cervidae, but was found in the European bison with a moderately high frequency, was also *A*. *sidemi* (44.6%). This blood-sucking nematode, which originates from Asia (mainly infecting the sika deer *Cervus nippon*), was noticed for the first time in the BPF European bison population in 2000 [49]. It reached 100% prevalence of infection within only a few years and remained at very high levels (prevalence ≥ 90%) for at least the next 10 years. Associated infection intensity was also very high, reaching over 40,000 *A. sidemi* specimens per one European bison individual [12]. In the European bison, which acted as a new host for this parasite, infections were very severe [12], which could lead to inflammation of the digestive system, chronic diarrhoea, anaemia, and even death, especially in young individuals [20, 50, 51]. Our molecular analysis confirms the trend recorded since 2011, related to the slight drop in the prevalence of *A. sidemi* infection in the studied population [12].

The tapeworm *M. benedeni* was found in the studied European bison population with the prevalence of 36.2%, which is slightly lower than that found almost 20 years ago, 41% [52], and in 2007–2011, 42.3% [53]. This may be related, among other things, to the rapid decrease in the number of small farms keeping cows since the 1990s [54], because *M*. *benedeni* is a common parasite of other ruminants, including cattle. Additionally, this Cestoda species is found with a much higher prevalence in young calves, not only in the European bison, with a prevalence up to 50% [18, 19, 53], but also in moose, where its prevalence reached 78% in this age class in Scandinavia [1]. In the BPF, fecundity and the proportion of the European bison calves in the population decreased over time (1960–2020), which is a density-dependent mechanism related to population growth [23], and may explain the decrease in the prevalence of *M. benedeni*.

In the present study, *P*. *cervi* DNA was recorded in 30.1% of samples. The percentage of *P. cervi*–positive European bison is within the range of the values stated in other studies, that is, 23–50 in BPF and Borki Forest [46, 52, 55]. The other Cervidae fluke species, *P. fasciolaemorpha*, was found in our study with 26.5% prevalence, which is much higher than described by Dróżdż et al. [46] who found this parasite only in 6% of studied European bison individuals. This parasite should be considered an invasive species, which spreads throughout the entire country with migrating deer, causing severe parasitosis [20]. It is worth noting that the greatest decrease in the prevalence of the studied parasites (almost 80%) was noted for *O*. *venulosum*, which was previously found in 100% of studied European bison individuals [56]. *H. contortus* and *T*. *hydatigena* were reported with the lowest prevalence. The former is a blood-sucking nematode found, among others, in the captive European bison in a breeding centre in BPF [57, 58], and Avesta, Sweden, with a prevalence of 55% [22], and it was also discovered in free-ranging European bison herds living in Russia and Romania [59, 60]. It is also worth mentioning that eggs of *A. sidemi* and *H. contortus* (both blood-sucking nematodes) are indistinguishable using coprological methods and can only be determined to the species level using the more accurate molecular methods [22]. Therefore, when coproscopic examinations are carried out, the results should be reported as gastrointestinal nematodes from the Trichostrongylidae family [21]. *T. hydatigena* is a widespread cestode that uses canids or felines as definitive hosts, while the larval stage appears in the peritoneal cavity of a wide variety of intermediate hosts, including horses; small ruminants (sheep, goats); and less frequently pigs, wild boar, or deer [61, 62]. Eggs of this tapeworm are passed with the faeces of canids and are occasionally ingested by intermediate hosts during grazing on contaminated pasture [63]. Accidentally swallowed eggs from which larvae hatch in the intestine may explain the presence of this tapeworm parasite DNA in the European bison faeces. On the other hand, in some intermediate hosts, such as the chamois (*Rupicapra rupicapra tatrica*) in Slovakia, larvae of *T*. *hydatigena* were found in the stomach [63]. The similar structure of the stomach in the chamois and the European bison may also explain the presence of DNA of this helminth in the studied population.

Supplementary feeding is widely used in conservation and wildlife management to support the survival and condition of animal populations and to reduce human–wildlife conflicts, but it may influence the use of space, causing aggregation of animals and an increase in the rate of intra- and interspecific interactions [28, 64, 65]. This may lead to abnormally high local population densities or host aggregations and levels of contact between them, which promotes the transmission of parasites within wildlife and between wildlife and domestic livestock [38, 66]. Hence, supplementary feeding is considered a controversial practice [67–69], despite being a widespread management practice of the majority of free-ranging populations of European bison, including those in the BPF [70]. Since parasitic diseases are one of the major threats to European bison [3], we decided to determine whether winter supplementary feeding of the European bison affects the endoparasite richness and parasite prevalence of selected cervid helminths. Out of the nine studied species of parasites, we found a statistically higher prevalence of three parasite species: *M. benedeni*, *P. fasciolaemorpha*, and *O. venulosum* in supplementary-fed European bison compared to nonfed ones and in one—*P. cervi*—the relation was opposite. Transmission of all these parasite species is waterborne, foodborne, or both, and it is known that European bison get infested with parasites through contact with contaminated water and pastures [71]. *M. benedeni* is a cosmopolitan tapeworm, with ruminants serving as definitive hosts and mites serving as intermediate hosts. Monieziosis transmission takes place when ruminants consume infected mites while grazing [53]. *P. fasciolaemorpha* is transmitted similarly, by consuming snails which act as intermediate hosts [72], and *O. venulosum* has a simple life cycle with eggs shedding into the environment and transmission by ingestion of infective larvae with the contaminated soil [73].

Interestingly, in our study, *P. cervi* was detected with a higher prevalence (42.5%) in nonfed European bison compared to supplementarily fed ones (18.6%), even though it has a similar transmission route as *P. fasciolaemorpha*, with snails also serving as intermediate hosts. A possible explanation is that nonfed European bison more often utilise meadows and areas outside the forest. This may promote paramphistomiasis transmission by both contact with livestock ruminants grazing in these areas and the presence of more wet habitats [72]. In a previous study from the BPF, a statistically significant relationship was found between the winter densities and winter home ranges of the European bison and both the intensity of blood-sucking nematode *A*. *sidemi* infection [6, 12, 28] and the prevalence of coccidia infection [13]. Additionally, in winter, a higher prevalence of excreted parasite eggs of the Trichostrongylidae family, *Aonchotheca* sp., *Moniezia* spp., and *Nematodirus* spp. was found in comparison to the snow-free period [21]. However, another study revealed that winter supplementary feeding of moose in Norway did not show any effect on the prevalence of gastrointestinal parasite species or the intensity of infection [65]. Our research shows that parasitological monitoring may be used as one of the hygiene indicators of winter feeding places and may be included in the long-term and noninvasive monitoring programs of wildlife populations.

Besides the prevalence and diversity of endoparasite species in the European bison, we also tested for parasite associations between coinfecting parasite species in the BPF population to determine whether endoparasite species co-occurrence differs from the expectations. Hosts are often infested by multiple parasite species, but it is unclear whether patterns of parasite co-occurrence are driven by parasite habitat requirements or parasite–species interactions [74]. Understanding the role of biotic interactions is particularly pertinent to investigating parasite communities sharing the same host communities and individuals, as the interactions among parasites—both competition and facilitation—may have far-reaching implications for parasite transmission and evolution [75]. In the present study, interactions among coinfecting parasite species tended to be generally random for the European bison population, but we found some evidence for both positive and negative associations, with positive associations being slightly more common. Positive associations indicate that either the infected host individuals are more susceptible to infection or infection by a parasite species facilitates another, leading to a positive parasite–parasite interaction [75, 76]. Helminth parasites can suppress the host's immune function, thus facilitating infection by other parasite species [77]. Two pairs of endoparasite species coinfecting the European bison population were negatively associated. These associations occurred between two parasite species of the *Ostertagia* genus, for which the highest prevalence was noted, and between a pair of parasite species inhabiting the digestive system of the host: fluke *P*. *cervi* and nematode *H*. *contortus*. Parasite associations are generally negative when parasite species infect the same host tissue, competing for both resources and space, which may be potentially modulated through the host immune response [75, 78, 79]. Associations between parasites depend on covariates related to geographic location, season, and host features. Understanding the factors influencing parasite colonisation, diversity, and species association patterns is important in parasite ecology research. This knowledge provides valuable information for the development of predictive models in disease ecology based on host traits and host–parasite or parasite–parasite interactions [80].

## 5. Conclusions

The European bison was recognised as a refugee species confined to nonoptimal forest habitats [70]. The developed molecular monitoring allowed us to assess parasite species composition and the prevalence of nine selected cervid helminths in the European bison population in the BPF, NE Poland. Our survey confirmed that European bison parasitofauna includes helminths transferred from deer. Red deer, roe deer, and moose share habitats and resources with the European bison, which increases the risk of host crossinfection. We also compared endoparasite species richness between supplementarily fed and nonfed European bison herds in the study area. This necessitates winter feeding leading to the concentration of European bison in a limited space, which increases the risk of parasite transmission. Introducing European bison to forest habitats, where there is insufficient food for such large herbivores in winter, leads to seasonal migration of the European bison to agricultural areas, where they have contact with cattle [38, 66], which may increase the risk of parasite transmission [75] and increase the prevalence and intensity of parasitic infections [12, 28]. We conclude that the conservation management of the European bison can significantly contribute to the spread of parasites from other hosts and a high level of parasitism observed in the species. Thus, the use of methods such as molecular monitoring that enable rapid and noninvasive scanning of the health status of animal populations is very important for the conservation of endangered species.

## Figures and Tables

**Table 1:** Prevalence of nine endoparasites obtained from molecular analysis of the European bison faecal samples.

**Parasite species**	**Fed herds (43 samples)**		**Nonfed herds (40 samples)**		**p**
	**No. of positive**	**% (CI)**	**No. of positive**	**% (CI)**	
*Moniezia benedeni*	20	46.5 (32.5–61.1)	10	25 (14.2–40.1)	**0.0415**
*Taenia hydatigena*	4	9.3 (3.7–21.6)	2	5 (1.4–16.5)	0.4495
*Parafasciolopsis fasciolaemorpha*	16	37.2 (24.4–52.2)	6	15 (7.1–29.1)	**0.0220**
*Paramphistomum cervi*	8	18.6 (9.7–32.6)	17	42.5 (28.5–57.8)	**0.0177**
*Oesophagostomum venulosum*	14	32.6 (20.5–47.5)	5	12.5 (5.5–26.1)	**0.0298**
*Ashworthius sidemi*	19	44.2 (30.4–58.9)	18	45 (30.7–60.2)	0.9406
*Haemonchus contortus*	8	18.6 (9.7–32.6)	7	17.5 (8.8–32.0)	0.8960
*Ostertagia antipini*	25	58.1 (43.3–71.6)	17	42.5 (28.5–57.8)	0.1545
*Ostertagia leptospicularis*	40	93.0 (81.3–97.6)	34	85 (70.9–92.9)	0.2401

*Note:* A significant difference between winter supplementarily fed and nonfed herds in the chi-square test is marked in bold.

Abbreviation: CI, 95% confidence interval.

## Data Availability

The data used to support the findings of this study are included within the article. If anyone is interested in the raw data (Excel), it is available from the corresponding author upon request.

## Supporting Information

Supporting Information 1 Table S1. The sequence of PCR primers with fluorescent dyes (FAM—blue, NED—black, PET—red, and VIC—green) used in molecular identification of endoparasite species occurring in the European bison faecal samples collected from the Białowieża Primeval Forest. –: parasite species absent in analysed samples.

Supporting Information 2 Table S2. Prevalence (in percent; CI, 95% confidence interval) of nine endoparasite species obtained from molecular analysis of the European bison faecal samples collected from 10 herds from the population in the Białowieża Primeval Forest. Supplementarily fed Herds 1, 2, 4, 6, and 10 are underlined. Nonfed herds are 3, 5, 7, 8, and 9; *N*, number of the European bison faecal samples.
