# Supplementary material for: Molecular Identification of Selected Cervid Helminths in Supplementarily Fed European Bison Population
Source: J Parasitol Res. 2024 Nov 9;2024:2600633. doi: 10.1155/2024/2600633 (PMC11568891; doi:10.1155/2024/2600633)
Supplement: Supporting Information 1 — Table S1. The sequence of PCR primers with fluorescent dyes (FAM—blue, NED—black, PET—red, and VIC—green) used in molecular identification of endoparasite species occurring in the European bison faecal samples collected from the Białowieża Primeval Forest. –: parasite species absent in analysed samples. [file 2600633.f1.docx]

Table S1: The sequence of PCR primers with fluorescent dyes used in molecular identification of endoparasite species occurring in the European bison faecal samples collected from the Białowieża Primeval Forest.

| **Parasite species** | **Gene** | **PCR Primers sequence 5’–3’** | **Product length (bp)** | **Fluorescent  dyes** | **Panel** | **Source** |
| --- | --- | --- | --- | --- | --- | --- |
| *Moniezia benedeni* | ITS-2 region | F-GACATCTTGAACGCATATTGCGGC R-TCAGGAGAAGCTACGACCCGATTC | 283 | FAM | II | Świsłocka et al. 2020 |
| *Echinococcus granulosus* | COX1 | F-CTCTGCATTTGGCTGGTGTTTCAAG R-TAGGATCACCACCACCTAACGGATC | 235 | PET | – | Świsłocka et al. 2020 |
| *Taenia hydatigena* | COX1 | F-GATGCCCATATTAATTGGTGGTT R-TGACGCACCAGCTAAATGCAACG | 252 | VIC | II | This study |
| *Parafasciolopsis fasciolaemorpha* | 28S rRNA | F-CCTGAGCAGACCTTGGAGTC  R-CAGACAAGCCGGACCTTTAC | 173 | NED | I | This study |
| *Paramphistomum cervi* | ITS-2 region | F-CTATCACGACGCCCAAAAAGTC R-CCAGTTACACTAACAAAGGCAC | 220 | NED | I | This study |
| *Elaphostrongylus alces* | ITS-2 region | F-CCTGATGTATCTAGCGCTTCTATGA  R-ATCACTAGCATAGGCACACATCATC | 200 | NED | – | Świsłocka et al. 2020 |
| *Elaphostrongylus cervi* | ITS-2 region | F-CGCGTCAAAACGATAGACGGAC  R-CAACATCACTAGCATAGATACGC | 156 | PET | – | Świsłocka et al. 2020 |
| *Parelaphostrongylus tenuis* | ITS-2 region | F-CTATGCTTCTCTATGTATGCGACGA R-TGGTAACAACATCACTAGCATAGGC | 192 | VIC | – | Świsłocka et al. 2020 |
| *Dictyocaulus capreolus* | COX1 | F-GACCTGATATGAGTTTTCCTCGT R-CCACCTAAAATAGATCTCAAACC | 213 | FAM | – | Świsłocka et al. 2020 |
| *Dictyocaulus cervi* | ITS-2 region | F-GCTGCATAATGTTGTCAAACGG R-ACTGCTTCTTTAGTCAACGATC | 195 | NED | – | Świsłocka et al. 2020 |
| *Ostertagia antipini* | ITS-1 region | F-GAGGTGTCTATGTATGACATGAGTCG R-ACCACCATTATAAAACTATGAGGCGG | 200 | FAM | I | Świsłocka et al. 2020 |
| *Ostertagia leptospicularis* | ITS-1 region | F-GATTACGTCCCTGCCCTTTGTA R-GTTTCTCATGATCAAAGGAACC | 228 | FAM | II | Świsłocka et al. 2020 |
| *Ashworthius sidemi* | ND4 | F-GGTGAGTATTATCACTCATGTTC  R-TAGTTCACATAATTCTATTGAC | 177 | VIC | II | This study |
| *Haemonchus contortus* | 18S rRNA | F-GCTCTATTACATGAGGTGTCTATGT R-TGTGAACACTAAGTTCTGCAAACTG | 286 | PET | I | This study |
| *Oesophagostomum venulosum* | ITS2 | F-GTCGAACGATGCTTGCACGT R-ACACTTTGACAGTGACAACC | 161 | VIC | I | This study |
| *Setaria tundra* | COX1 | F-CTCCTGAAATAGCTTTTCCTCGTG R- AGTTCAAACAAACATACTAGCCTG | 292 | PET | – | This study |

FAM – blue; NED – black; PET – red; VIC – green; – parasite species absent in analysed samples.
