# Supplementary material for: Molecular Identification of Selected Cervid Helminths in Supplementarily Fed European Bison Population
Source: J Parasitol Res. 2024 Nov 9;2024:2600633. doi: 10.1155/2024/2600633 (PMC11568891; doi:10.1155/2024/2600633)
Supplement: Supporting Information 2 — Table S2. Prevalence (in percent; CI, 95% confidence interval) of nine endoparasite species obtained from molecular analysis of the European bison faecal samples collected from 10 herds from the population in the Białowieża Primeval Forest. Supplementarily fed Herds 1, 2, 4, 6, and 10 are underlined. Nonfed herds are 3, 5, 7, 8, and 9; N, number of the European bison faecal samples. [file 2600633.f2.docx]

Table S2: Prevalence (in %; CI – 95% confidence interval) of 9 endoparasite species obtained from molecular analysis of the European bison faecal samples collected from 10 herds from population in the Białowieża Primeval Forest. Supplementarily fed herds: 1, 2, 4, 6, and 10 are underlined; Non-fed herds are: 3, 5, 7, 8 and 9; N – number of the European bison faecal samples.

| **Parasite   species**  **Herds** | **N** | ***Moniezia benedeni* % (CI)** | ***Taenia hydatigena* % (CI)** | ***Parafasciolopsis fasciolaemorpha* % (CI)** | ***Paramphistomum cervi* % (CI)** | ***Ostertagia antipini* % (CI)** | ***Ostertagia***  ***leptospicularis* % (CI)** | ***Ashworthius sidemi* % (CI)** | ***Haemonchus contortus* % (CI)** | ***Oesophagostomum venulosum* % (CI)** |
| --- | --- | --- | --- | --- | --- | --- | --- | --- | --- | --- |
| **Herd 1** | 10 | 50 (23.66–76.34) | 20  (5.67–50.98) | 30  (10.78–60.32) | 20  (5.67–50.98) | 50 (23.66–76.34) | 90  (59.59–98.21) | 30  (10.78–60.32) | 30  (10.78–60.32) | 40 (16.82–68.73) |
| **Herd 2** | 8 | 62.5  (30.58–86.31) | 12.5  (2.24–47.09) | 87.5  (52.91–97.76) | 25  (7.15–59.07) | 50  (21.52–78.48) | 87.5  (52.91–97.76) | 75  (40.93–92.85) | 12.5  (2.24–47.09) | 37.5 (13.69–69.42) |
| **Herd 3** | 10 | 30  (10.78–60.32) | 0  (0–27.75) | 0  (0–27.75) | 50  (23.66–76.34) | 20  (5.67–50.98) | 90  (59.59–98.21) | 60  (31.27–83.18) | 30  (10.78–60.32) | 0  (0–27.75) |
| **Herd 4** | 12 | 41.67  (19.33–68.05) | 8.33  (1.49–35.39) | 16.67  (4.70–44.80) | 33.33  (13.81–60.94) | 66.67  (39.06–86.19) | 91.67  (64.61–98.51) | 50  (25.38–74.62) | 8.33  (1.49–35.39) | 25 (8.89–53.23) |
| **Herd 5** | 4 | 50 (15–85) | 0  (0–48.99) | 0  (0–48.99) | 75  (30.07–95.44) | 25  (4.56–69.93) | 75  (30.07–95.44) | 25  (4.56–69.93) | 50 (15–85) | 50 (15–85) |
| **Herd 6** | 8 | 50 (21.52–78.48) | 0  (0–32.44) | 37.5 (13.69–69.42) | 0  (0–32.44) | 50  (21.52–78.48) | 100  (67.56–100) | 12.5  (2.24–47.09) | 0  (0–32.44) | 37.5 (13.69–69.42) |
| **Herd 7** | 7 | 14.29  (2.57–51.31) | 28.57  (8.22–64.10) | 57.14  (25.05–84.18) | 57.14  (25.05–84.18) | 28.57  (8.22–64.10) | 100  (64.57–100) | 28.57  (8.22–64.10) | 14.29  (2.57–51.31) | 42.86 (15.82–74.95) |
| **Herd 8** | 7 | 42.86  (15.82–74.95) | 0  (0–35.43) | 42.86 (15.82–74.95) | 14.29  (2.57–51.31) | 100  (64.57–100) | 100  (64.57–100) | 57.14  (25.05–84.18) | 0  (0–35.43) | 0  (0–35.43) |
| **Herd9** | 12 | 8.33  (1.49–35.39) | 0  (0–24.25) | 0  (0–24.25) | 33.33  (13.81–60.94) | 41.67  (19.33–68.05) | 66.67  (39.06–86.19) | 41.67  (19.33–68.05) | 33.33  (13.81–60.94) | 8.33 (1.49–35.39) |
| **Herd 10** | 5 | 20  (3.62–62.44) | 0  (0–43.45) | 0  (0–43.45) | 0  (0–43.45) | 80  (37.56–96.38) | 100  (56.55–100) | 60  (23.07–88.24) | 0  (0–43.45) | 0  (0–43.45) |
| **All** | 83 | 36.15  (26.64–46.88) | 7.23  (3.36–14.89) | 26.51  (18.20–36.89) | 30.12  (21.31–40.69) | 50.60  (40.06–61.09) | 89.16  (80.66–94.19) | 44.58  (34.36–55.27) | 18.07  (11.27–27.70) | 22.89 (15.17–33.01) |
